# The Development of CMOS Amperometric Sensing Chip with a Novel 3-Dimensional TiN Nano-Electrode Array

**DOI:** 10.3390/s19050994

**Published:** 2019-02-26

**Authors:** Chun-Lung Lien, Chiun-Jye Yuan

**Affiliations:** 1 Department of Biological Science and Technology, National Chiao Tung University, Hsinchu 30068, Taiwan; andrew0958.bt99g@g2.nctu.edu.tw; 2 Institute of Molecular Medicine and Bioengineering, National Chiao Tung University, Hsinchu 30068, Taiwan; 3 Center for Intelligent Drug Systems and Smart Bio-Devices (IDS2B), National Chiao Tung University, Hsinchu 30010, Taiwan

**Keywords:** nano-electrode, CMOS, 3D Sensor, titanium nitride, hydrogen peroxide

## Abstract

An electrochemical sensing chip with an 8 × 8 array of titanium nitride three-dimensional nano-electrodes (TiN 3D-NEA) was designed and fabricated via a standard integrated complementary metal oxide semiconductor process. Each nano-electrode in 3D-NEA exhibited a pole-like structure with a radius of 100 nm and a height of 35 nm. The numeric simulation showed that the nano-electrode with a radius of around 100 nm exhibited a more uniformly distributed electric field and a much higher electric field magnitude compared to that of the microelectrode. Cyclic voltammetry study with Ru(NH_3_)_6_^3+^ also revealed that the TiN 3D-NEA exhibited a much higher current density than that obtained from the microelectrode by two orders of magnitude. Further studies showed that the electrocatalytical reduction of hydrogen peroxide (H_2_O_2_) could occur on a TiN 3D-NEA-based sensing chip with a high sensitivity of 667.2 mA⋅mM^−1^⋅cm^−2^. The linear detection range for H_2_O_2_ was between 0.1 μM and 5 mM with a lowest detection limit of 0.1 μM. These results indicated that the fabricated TiN 3D-NEA exhibited high catalytic activity and sensitivity to H_2_O_2_ and could be a promising sensor for H_2_O_2_ measurement.

## 1. Introduction

Electrochemical sensing technology has been widely adopted in the design of biosensors for clinical diagnosis and point-of-care (POC) testing due to its simple design, quick detection, and cost effectiveness [1]. Many methods have been developed to improve the electrochemical reactivity and sensitivity of electrodes, including anodic surface cleaning [2], surface modification [3,4], plasma treatment [5], and miniaturization [6,7,8,9,10]. Among these methods, miniaturization exhibits several advantages, including low power consumption, small sample volume requirement, high electrochemical responses, low electric noise, and portability. Small sample volume requirement is helpful in the development of biomedical devices for detecting rear or dangerous biological samples. When the electrode size is reduced to nano-scale, the diffusion becomes convergent to the surface of the electrode and enhances the mass transport of electroactive species to its surface [11,12,13,14,15,16]. Hence, the electrochemical responses on the nano-electrode may not be limited by the diffusion of electroactive species due to the high mass transport rate [6,13,14]. Accordingly, the responses on nano-electrodes may reflect the actual enzymatic activity or chemical reactions during the analysis.

Electrode miniaturization may have some drawbacks, such as reduced total surface area, reduced biomolecule conjugation, and limited reactive sites. These problems may further lead to low electrochemical responses and quick saturation during detection. To increase the total surface area and reduce the influence of the miniaturization on the performance of electrodes, generating the electrode array [6,7,8,9,10,11,12,13,17,18,19,20,21,22] and/or forming the protruding nano-structures through post-fabrication modification [17,23,24,25,26,27,28,29,30,31] are usually adopted for the design of micro- and nano-electrodes. However, most of the reported micro- and nano-electrode arrays with or without protruding structures were fabricated by microelectromechanical system (MEMS)/nanoelectromechanical system (NEMS) or MEMS/NEMS integrated with the complementary metal oxide semiconductor (CMOS) technology. The integration of MEMS and CMOS processes opens up new possibilities in the design and development of various micro-sensors and electrodes. However, the need for dedicated manufacturing foundries, high cost, and limited choices of materials, design rules, and micromachining processes may be the limitations for the fabrication of sensors and actuators by integrating MEMS and CMOS fabrication technologies. With these limitations, the standard CMOS process may a good choice to generate sensors and electrodes with simple nano-configurations or three-dimensional (3D) nano-structures. With the standard CMOS process, the fabrication of nano-scaled electrodes can be controlled by the well-developed processes such as photolithography, chemical vapor deposition, and e-beam lithography. Hence, the mass production of nano-sensors with highly uniform 3D structure and well-defined geometry is feasible. However, the material suitable for the fabrication of the biosensing chip is limited in the standard CMOS process. Among materials frequently used in the CMOS process, titanium nitrite (TiN), a ceramic material, exhibits an excellent combination of chemical, physical, mechanical, and electrical properties such as high hardness, good chemical stability, abrasion and corrosion resistance, chemical inertness, high conductivity, and biocompatibility [23,32,33,34,35]. Most importantly, compared to Au, Pd, or Pt, TiN is more cost-effective and less sticky to the reaction chambers without causing contamination. Accordingly, TiN is regularly used in the standard CMOS process for the fabrication of the CMOS devices [24,36,37]. Hence, even though the electric conductivity of TiN (8.69 × 10^6^ Ω^−1^·m^−1^) is about 20.9% of that of gold (4.17 × 10^7^ Ω^−1^·m^−1^), it is suitable for the fabrication of a sensing chip via the standard CMOS process.

In this work, an electrochemical sensing chip with TiN-based 3D nano-electrode array (TiN 3D-NEA) was proposed to be fabricated by the standard CMOS process. The 3D configuration can effectively increase the surface area of nano-electrodes, providing more space for bioconjugation and generating more reactive sites for reaction [11,12,13,17,38,39]. Subsequently, the redox responses and the electrochemical behavior of the fabricated CMOS 3D-NEA chip was investigated by cyclic voltammetry, and it was found that the 3D-NEA sensing chip exhibited a voltammetry response deviating from the conventional diffusion-controlled current flow. Furthermore, hydrogen peroxide (H_2_O_2_) was found to be electrocatalytically reduced on the CMOS 3D-NEA sensing chip with high sensitivity and a wide linear range of detection.

## 2. Materials and Methods 

### 2.1. Materials

Phosphate buffer solutions (PBS) with different pH were prepared from 0.1 M KH_2_PO_4_ and 0.1 M K_2_HPO_4_. Ascorbic acid (AA), uric acid (UA), dopamine (DA), acetaminophen (AC), hexaammineruthenium (III) chloride (Ru(NH_3_)_6_Cl_3_), and glucose were purchased from Sigma Aldrich. Ultra-pure water with resistance higher than 18 M·ohm·cm was employed to prepare all solutions and electrolytes. All other reagents were analytical grade.

### 2.2. Chip Design and Fabrication

**Figure 1:**
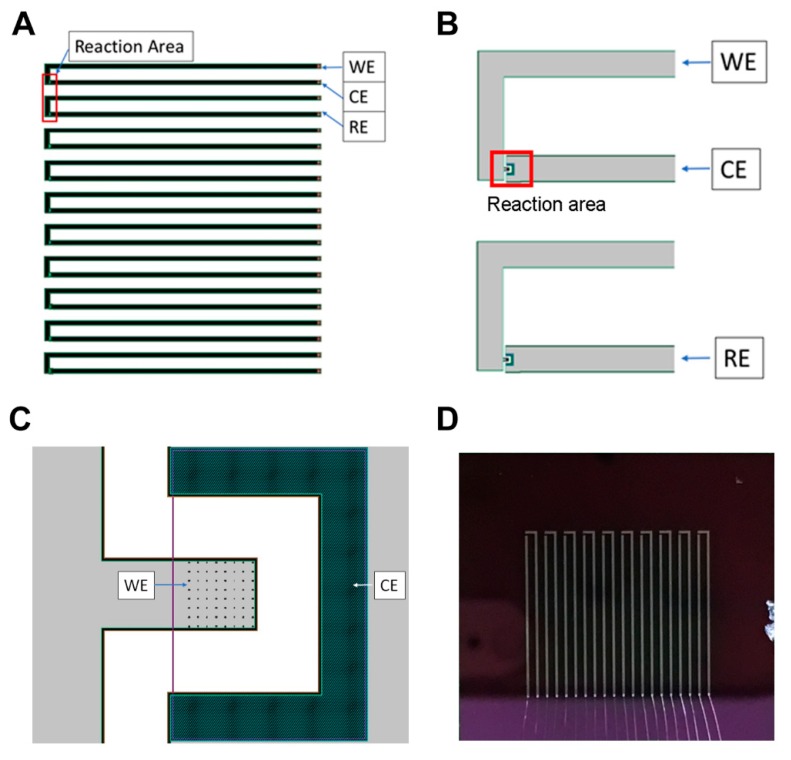
The complementary metal oxide semiconductor (CMOS) sensing chip with titanium nitride three-dimensional nano-electrodes (TiN 3D-NEA). (**A**) The sensing chip contains 10 units of working (WE) and counter electrodes (CE). (**B**) The enlarged reaction area of the electrode set in (A). The counter electrode of the adjacent electrode unit was used as the reference electrode (RE). (**C**) The diagram of an electrochemical sensing unit on the CMOS chip. Each sensing unit contained an 8 × 8 nano-electrode array as a WE and a CE. (**D**) The fabricated CMOS sensing chip (20 mm × 20 mm) with bonding wires (bottom part).

The sensing chip (20 mm × 20 mm) containing 10 sensing units (Figure 1A,D), which were connected to their own contact pads through connecting wires, was fabricated via a standard CMOS process in the National Nano Device Laboratories (NDL) at the Science-Based Industrial Park, Hsinchu, Taiwan.

**Figure 2:**
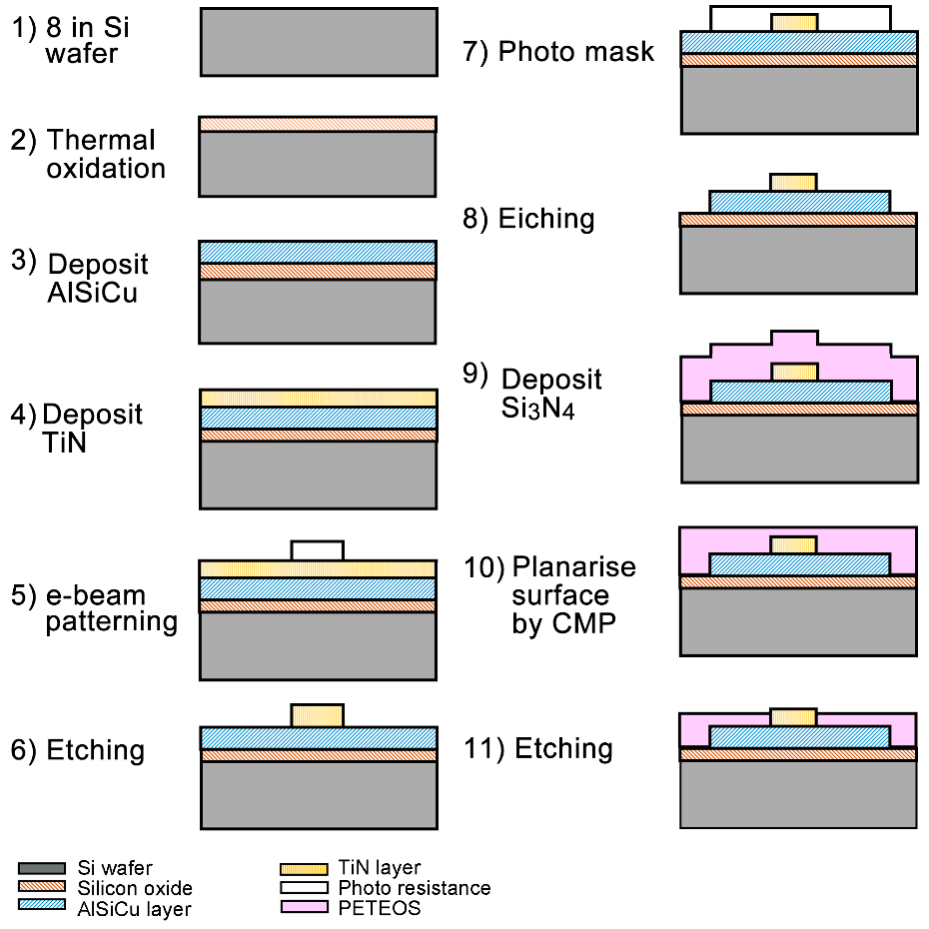
Fabrication procedures of standard CMOS process to construct a sensing chip with 3D-NEA.

The fabrication flow for 3D NEA is illustrated in Figure 2. Briefly, an 8-inch single crystalline silicon wafer (step 1) with a p-type (100)-oriented substrate that exhibited a resistance of 0.5–100 Ω·cm and a thickness of 725 μm was used for the fabrication of a sensing chip containing 3D NEA. The fabrication was started with the thermal oxidation of the silicon wafer to generate a 1500 Å thick silicon dioxide substrate (step 2) for electrical isolation between the substrate and the surface. On the silicon dioxide, a 4000 Å thick AlSiCu adhesion layer (step 3) as adhesive as well as conducting layer and a layer of 2000 Å thick TiN (step 4) as an electrode material were sequentially deposited by physical vapor deposition (PVD). The conductivity between AlSiCu and TiN was enhanced by the inductively coupled plasma reactive ion etching process at the interface to remove the possible metal oxides generated during the fabrication process. After spin-coating the negative e-beam photoresistor (PR), the pattern of the working electrode (WE) and the counter electrode (CE) were generated by e-beam lithography to provide precise control of electrode size and shape (step 5). The patterned TiN electrodes, including WE and CE, were then exposed by wet etching (step 6). The wider width AlSiCu metal pattern underneath the TiN layer was generated by a photomask containing the pattern for the interconnections and bonding pads to connect the TiN electrodes to the bonding pad (steps 7 and 8). After etching and removing the PR, the surface of the patterned wafer was passivated by a layer of Si_3_N_4_ using plasma enhanced tetraethylorthosilicate (PETEOS) method (step 9) to cover the spaces between electrodes. This Si_3_N_4_ passivation could prevent the background current and enhance the electric field on the exposed surface of the 3D-NEA. Next, the surface of the wafer was planarized by the chemical-mechanical planarization (CMP) process (step 10) followed by etching the Si_3_N_4_ layer to expose the 3D-NEA and CE, interconnections and bonding pads (step 11). The structure of the fabricated CMOS sensing chip (Figure 1D) was monitored and analyzed on the Helios Nanolab 660 Dual Beam focused ion beam (FIB) (FEI Co., Hillsboro, OR, USA) by the Integrated System Technology Inc. (Hsinchu, Taiwan).

### 2.3. Apparatus and Electrochemical Measurement

The CMOS sensing chip was fixed on a printed circuitry board (PCB) and wire bonded by the Silicon Application Corp. (Hsinchu, Taiwan) (Figure S1 in the supplementary material) to develop a TiN 3D-NEA-based sensing system, which was then connected to the CHI6116E electrochemical potentiostat (CH Instruments, Inc., Austin, TX, USA) and a personal computer to perform the electrochemical measurements. The counter electrode of the adjacent sensing unit was used as the reference electrode (RE) in the electrochemical measurements (Figure 1B). The cyclic voltammetry (CV) was performed in the phosphate buffer (25 mM Na_2_HPO_4_ and 25 mM NaCl, pH 7.0) containing 5.0 mM Ru(NH_3_)_6_Cl_3_ in a potential range between −0.6 V and +0.3 V versus RE at the scan rates of 20, 50, 100, and 200 mV/s. The electrochemical measurements of H_2_O_2_ on the TiN 3D-NEA-based sensing system at various concentrations were performed by CV in the phosphate buffer at the scan rate of 100 mV/s in a potential range of –0.50 V~0 V versus RE.

## 3. Results

### 3.1. Numeric Simulation of the Electric Field Distribution of Nano- and Micro-Electrodes 

**Figure 3:**
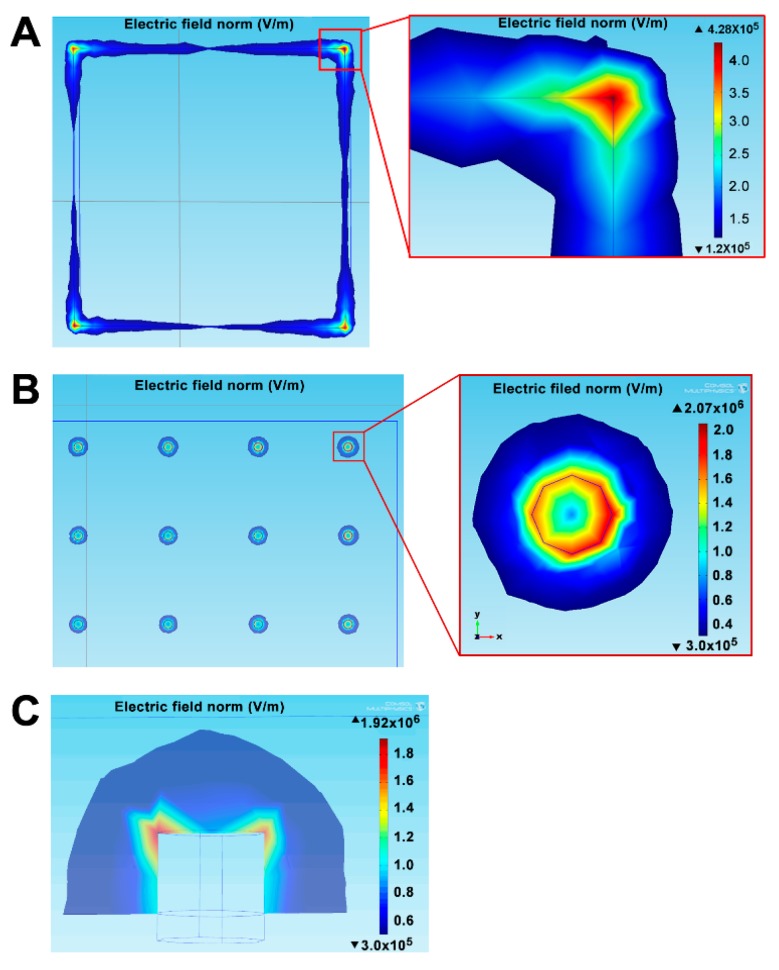
Simulation of electric field distribution of three different of electrodes. (**A**) Electric field distribution of the 7.4 × 7.4 μm^2^ microelectrode; (inset) the enlarged image of the electric field distribution at the corner of microelectrode. (**B**) Electric field distribution of 3D-NEA with a radius of 0.1 μm and a height of 0.1 μm; (inset) the electric field distribution for a single nano-electrode with a radius of 0.1 μm. (**C**) The cross-section view of the electric field distribution for the 3D nano-electrode with a radius of 0.1 μm. These data were simulated by the COMSOL Multiphysics software (v.4.4).

The electric field strength and distribution of the conventional microelectrode and the proposed nano-electrode array were compared by numeric simulation via the simulator (COMSOL MULTI PHYSICS) (Figure S2). The maximum electric field (4.28 × 10^5^ V m^−1^) of the micro-electrode with a size of 7.4 × 7.4 μm^2^ (the surface area of 5.48 × 10^−7^ cm^2^) appeared only at four corners, while the electric field strength on the surface and at four sides of the microelectrode was minimal (Figure 3A). In comparison, the proposed 8 × 8 array of 3D nano-electrodes with a radius of 0.1 μm and a height of 0.1 μm (the estimated surface area of 9.42 × 10^−10^ cm^2^ for each nano-electrode) exhibited a more evenly distributed electric field on the edge of the nano-electrode with a maximum magnitude of 2.07 × 10^6^ V·m^−1^, which was about five times higher than that on the microelectrode (Figure 3B, inset). This result suggests that the electric field of the electrode can be greatly enhanced by reducing its size from micro- to nano-scale. The reduction of the surface area may not only enhance the electric field but also reduce the electric double layer impedance and the noise at the electrode/electrolyte interface [20,40,41].

Interestingly, the strong electric field could be seen not only at the upper end of the 3D nano-electrode but also on its side wall (Figure 3C). Upon the simulation, about 75% of the maximal electric field strength could be retained on the side wall at 0.04 μm below the upper end, whereas about 56.8% of the maximal electric field strength could be observed at the position 0.1 μm below the upper end. This result suggests that more surface area on the 3D nano-electrode can be covered by the high electric field than that on the planar nano-electrodes. Accordingly, the mass transport and redox reactions of electroactive species may be facilitated on or around the 3D nano-electrodes [18].

### 3.2. Fabrication and Characterizations of Nano-Electrode Array

**Figure 4:**
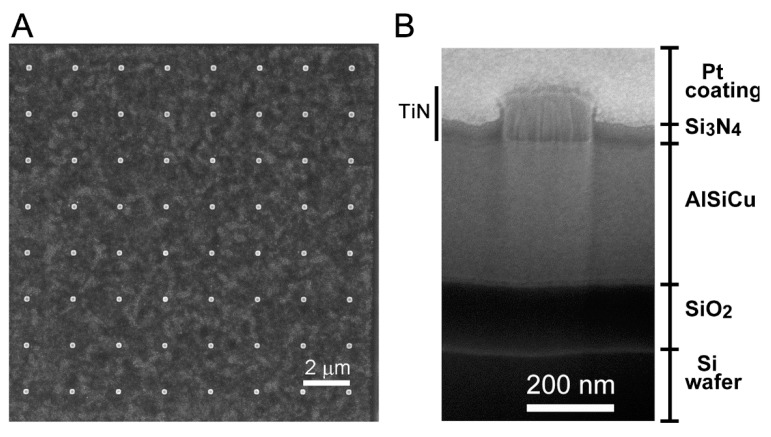
SEM images of 3D-NEA and the single nano-electrode. (**A**) The top view of 3D-NEA. (**B**) The focused ion beam (FIB) image of the cross section view of the nano-electrode in the 3D-NEA with a radius of 0.1 μm. The organization and distribution of Si wafer, SiO_2_, AlSiCu, TiN electrode, and Si_3_N_4_ structures in the CMOS chip are indicated on both sides.

The CMOS sensing chip containing 10 sensing units (Figure 1A,D) was fabricated following a procedure flow as illustrated in Figure 2. Each sensing unit contained a 3D-NEA as the WE and a CE (Figure 1C and Figure 4A). Each nano-electrode in the 3D-NEA exhibited the pole-like structure with a dimension of 0.1 μm in radius and 35 nm in height (Figure 4B). The FIB image of the cross-section of a 3D nano-electrode showed the sequential organization of Si wafer, SiO_2_, AlSiCu, TiN electrode, and Si_3_N_4_ structures from the bottom to the top of the device (Figure 4B). Pt coating on the top was used to protect the surface of the sample from the cutting effect during the FIB imaging. The results indicate that the fabrication of the 3D-NEA-based CMOS sensing chip was successful.

**Figure 5:**
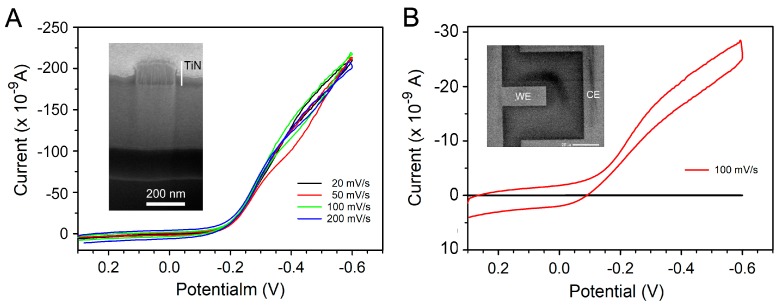
Cyclic voltammograms of Ru(NH_3_)_6_^3+^ on different electrodes. (**A**) 5 mM Ru(NH_3_)_6_^3+^ at 3D-NEA. (**B**) 5 mM Ru(NH_3_)_6_^3+^ at the microelectrode (33.6 × 8.8 μm^2^). The inset in each panel is the SEM image of the corresponding microelectrode or nano-electrode. The scale bar in the inset of panel (**B**) is 20 μm.

As a control, the microelectrode with a size of 33.6 × 8.8 μm^2^ was also fabricated by the standard CMOS process (Figure 5B, inset). The center-to-center distance between nano-electrodes of the 3D-NEA (Figure 4A) was 2 μm. Compared to the size of the nano-electrode, the distance between adjacent electrodes was much far away, making each nano-electrode behave as an individual sensing unit. With this design, the nano-electrodes in the 3D-NEA were assumed to have their own depletion zone without overlapping with others [15,42,43].

### 3.3. Electrochemical Characterization of TiN 3D-NEA-Based Sensing Chip

The electrochemical properties of the single sensing unit in the sensing chips with the 3D-NEA (the estimated total active area of 3.42 × 10^−8^ cm^2^) and microelectrode (the estimated active area of 2.96 × 10^−6^ cm^2^) were studied by CV in the presence of 5 mM Ru(NH_3_)_6_^3+^ in a potential range between −0.6 V and +0.3 V at the scan rates of 20, 50, 100, and 200 mV/s. Interestingly, the cyclic voltammogram of Ru(NH_3_)_6_^3+^ at different scan rates (from 20 mV/s to 200 mV/s) was nearly identical and exhibited steady state or quasi-steady state reaction curves on the sensing chip with 3D-NEA (the radius of nano-electrode ≤ 100 nm) (Figure 5A) [15,44]. The occurrence of this phenomenon may have been due to the fast mass transport of redox couple in the diffusion layer of the 3D-NEA [11,12,13,15,44], presumably driven by convergent diffusion near the electrode. Under these circumstances, the concentration gradient of electroactive species in the diffusion layer remained high and did not deplete significantly at different scan rates.

In comparison, the Ru(NH_3_)_6_^3+^ exhibited a typical cyclic voltammogram on the microelectrode with a limited current response (Figure 5B), presumably due to the planar diffusion near the microelectrode [15,42,43].

In addition to the electrochemical behavior, we found that the current densities obtained from two types of electrodes were inversely proportional to their surface areas (Table 1). The normalized current density of the 3D-NEA at −0.45 V and scan rate of 100 mV/s was 8.02 × 10^5^ A cm·mol^−1^, which was about two orders of magnitude higher than that calculated from the microelectrode (1.69 × 10^3^ A·cm·mol^−1^) (Table 1). The high current density observed in the 3D-NEA may have resulted in part from the radial diffusion and high electric field near the electrode, leading to the enhancement of electrochemical reactivity of the redox species. The calculated current density in the 3D-NEA was about 59.0% of that in the previously reported Au nano-electrode array with a small radius [11]. It may have been due to the low conductivity of TiN, which is about one fifth that of Au.

### 3.4. Electrochemical Measurement of H_2_O_2_

**Figure 6:**
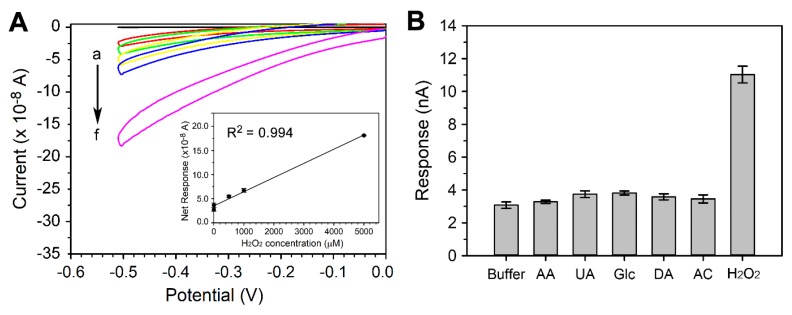
The electrochemical responses of H_2_O_2_ on CMOS sensing chip. (**A**) The cyclic voltammograms of various concentrations of H_2_O_2_ (a, no H_2_O_2_; b, 0.1 μM; c, 1 μM; d, 500 μM, e, 1 mM; f, 5 mM) on sensing chip with TiN 3D-NEA. The cyclic voltammetry (CV) was performed in a potential range of 0 V~−0.5 V at the scan rate of 100 mV/s. (inset), the linear regression plot of current responses versus H_2_O_2_ concentrations. (**B**) Responses of various interferants on the 3D-NEAL. The response of different interferants including 1 mM AA, 1 mM UA, 1 mM Glucose, 1 mM DA, 1 mM AC, and 1 mM H_2_O_2_ was performed in a potential rang of 0 V~−0.7 V at a scan rate of 100 mV·s^−1^. The response of these reagents at −0.7 V was then recorded and analyzed. The response of the phosphate buffer solution (PBS) was used as a negative control (buffer). The data are presented as mean ±S.D.

H_2_O_2_ is one of the products of the metabolism, enzyme reactions, and biological processes in living organisms [45,46]. The level of H_2_O_2_ is often used as an indicator of the physiological and pathological conditions of the organisms [47,48]. Therefore, H_2_O_2_ is an important target in clinical diagnosis, food analysis, environmental monitoring, and drug screening. In this study, the capability of the TiN 3D-NEA-based sensing chip to detect H_2_O_2_ was explored. The CV of various concentrations of H_2_O_2_ (0.1 μM, 1 μM, 0.5 mM, 1 mM, and 5 mM) was performed in PBS buffer with a pH of 7.4 in a potential range of –0.5 V ~ 0 V versus RE at a scan rate of 100 mV/s. H_2_O_2_ was found to exhibit the concentration-dependent reduction responses on the TiN 3D-NEA sensing chip (Figure 6A) with a high sensitivity (667.2 mA⋅cm^−2^⋅mM^−1^ H_2_O_2_). Besides, the electric noise of the 3D NEA chip was low (Figure 6A), and therefore the electrochemical responses of H_2_O_2_ could be measured by simply using the commercial potentiostat. The linear range of detection for H_2_O_2_ was determined between 0.1 μM and 5000 μM with a lowest detection limit of 0.1 μM (Figure 6A, inset). Compared to other micro- or nano-electrodes reported previously, the CMOS sensing chip developed in this study exhibited higher sensitivity and a wide linear range of detection toward H_2_O_2_ (Table 2) [19,20,23,49,50,51,52,53,54].

The detection of H_2_O_2_ in biological samples is usually interfered with the components in serum, such as AA, UA, DA, AC, and glucose. Hence, the electrochemical responses of these interferants were investigated on the TiN 3D-NEAL (Figure S3). The CV of 1 mM each of AA, UA, Glucose, DA, and AC in 0.1 M PBS, pH 7.4 was performed in a potential range of −0.7 V~0 V at a scan rate of 100 mV/s. Subsequently, the net current response of various interferants at −0.7 V was analyzed. As shown in Figure 6B, the AA, UA, Glucose, DA, and AC exhibited the electrochemical response equivalent to that of buffer only (3.08 ± 0.19 nA). In comparison, the response of 1 mM H_2_O_2_ on 3D NEAL was 11.03 ± 0.51 nA. This result suggests that H_2_O_2_ can be specifically detected on the developed CMOS sensing chip without the interference of the possible interferants in the biological samples. This characteristic is significant since the most essential challenge of non-enzymatic biosensors is to maintain the specificity that is usually conferred by enzymatic reactions.

## 4. Conclusions

In this study, a CMOS-based electrochemical sensing chip with the TiN 3D-NEA was proposed and fabricated by a standard CMOS process. The 3D-NEA was an 8 × 8 array of nano-electrodes with a radius of 0.1 μm and a spacing between electrodes of 2 μm. This design allowed the nano-electrode to behave as an individual sensing unit and have its own depletion zone. TiN was adopted for the electrode material because of its good chemical stability, chemical inertness, and biocompatibility. Most importantly, it could be fully integrated into the standard CMOS process with which the mass production of highly uniform, nano-scaled electrochemical sensing chips with well-defined geometry became easy. Furthermore, the 3D-NEA exhibited many electrochemical characteristics that were superior to those of conventional micro-electrodes. The CMOS sensing chip with the 3D-NEA also exhibited high sensitivity in detecting H_2_O_2_ and a wide linear range of detection. These results suggest that the fabricated CMOS sensing chip with the 3D-NEA exhibits great potential in the development of miniaturized, portable, and affordable biomedical devices for clinical diagnosis and POC testing in the future.

## Figures and Tables

**Table 1:** The comparison of the physical and electrochemical characteristics of electrodes with different geometries.

Electrodes	Electrode Radius ^a^ (μm)	Total Active Area ^b^ (cm^2^)	Current Density (A·cm·mol^−1^)
3D-NEA	0.10	3.42 × 10^−8^	8.02 × 10^5^
Microelectrode	(33.6 × 8.8) ^c^	2.96 × 10^−6^	1.69 × 10^3^

^a^ The shape of nano-electrodes is close to a cylinder, while the microelectrode is a rectangular. ^b^ The WE in 3D-NEA is an 8 × 8 array. Hence, the total active area is the sum of the surface area of 64 nano-electrodes. ^c^ The shape of microelectrode is rectangular.

**Table 2:** The comparison of the reactivity of 3D-NEA and the nanomaterial modified electrodes to H_2_O_2_.

Electrodes	Sensitivity (μA·mM^−1^·cm^−2^)	Linear Range of Detection (mM)	LOD (μM)	Reference
MCP/Pt/NPs	1280–1750	~7	9.6–17.7	[49]
PtNPs/PtME	NA	0.4–800	0.2	[50]
Cu_3_N NA/CF	7600	0.0001-10	0.0089	[20]
Ppy/GENS/Au	32	1–10	300	[51]
Au microelectrodes	NA	100–700	10	[52]
AgNW	1640	1.7–3.4	46	[53]
AgNW Array	NA	0.5–5.6	334	[19]
Carbon fiber microelectrodes	NA	2–2000	2	[54]
TiN NRA film with porosity	NA	0.02–3	20	[23]
3D TiN nano-electrode array	667200	0.0001–5	0.1	This work

LOD: limit of detection; CNT: carbon nanotube; NP: nanoparticle; MCP: micro carbon pillars; NA: nanowires array; CF: copper foam; Ppy: Polypyrrole; GENS: graphene nanosheets; NW: Nanowire; PtME: Pt micro-disk electrode; NRA: Nanorod arrays.

## Supplementary Materials

The following are available online at https://www.mdpi.com/1424-8220/19/5/994/s1, Figure S1: The CMOS sensing chip was fixed on a print circuitry board (PCB); Figure S2: Simulation of electric field distribution of microelectrode and nano-electrode array; Figure S3: The SEM imaging of the CMOS chip with 3D-NEAL.
