# Supplementary material for: The Development of CMOS Amperometric Sensing Chip with a Novel 3-Dimensional TiN Nano-Electrode Array
Source: Sensors (Basel). 2019 Feb 26;19(5):994. doi: 10.3390/s19050994 (PMC6427664; doi:10.3390/s19050994)
Supplement: Supplementary file 1 [file sensors-19-00994-s001.pdf]

sensors

MDPI

Article

The Development of CMOS Amperometric Sensing Chip with a Novel 3-Dimensional TiN Nanoelectrode Array

Chun-Lung Lien [1] **and Chiun-Jye Yuan** [1,2,3,*]

1 Department of Biological Science and Technology, National Chiao Tung University, Hsinchu, 30068 Taiwan; andrew0958.bt99g@g2.nctu.edu.tw
2 Institute of Molecular Medicine and Bioengineering, National Chiao Tung University, Hsinchu, 30068 Taiwan
3 Center for Intelligent Drug Systems and Smart Bio-devices (IDS²B), National Chiao Tung University, Hsinchu, 30010 Taiwan
* Correspondence: cjyuan@mail.nctu.edu.tw; Tel: +886-3-5731735; Fax: +886-3-5729288

Received: 19 December 2018; Accepted: 22 February 2019; Published: 26 February 2019

Simulation of electric field distribution

The simulation of the electrical field distribution of 3D NEA and microelectrode was carried out using COMSOL Multiphysics software. Under static conditions the electric potential, V, is defined by the equation below:

$$E = -\nabla V \tag{1}$$

When stationary electric currents in conductive media the stationary equation of continuity is considered. In a stationary coordinate system, the point form of Ohm's law states that:

$$J = \sigma E + J_e \tag{2}$$

Where, σ is the electrical conductivity (SI unit: S/m), and J_e is an externally generated current density (SI unit: A/m^2). The current sources Q_j (SI unit: A/m^3) can be generalize by the equation:

$$-\nabla\cdot(\sigma\nabla V - J_e) = Q_j \tag{3}$$

This equation accounts for charge migrations due to the applied voltage, V, at electrodes. Using above equations, the COMSOL simulations are able to calculate the electric field distribution in different electrode systems. Prior to the simulation, the parameters were set as follows: diffusion coefficient (D_o) for $Ru((NH_3)_6^{3+}$ = 7.09×10^{-10} $m^2{\cdot}s^{-1}$ [1]; applied potential (V_{app} DC) = 0.22 V; applied perturbation potential (V_{app} AC) = 5 mV; double layer capacitance (C_{dl}) = 20 $\mu F{\cdot}cm^{-2}$; Initial redox electrolyte concentration (C_o^*) =1 $mmol{\cdot}L^{-1}$; heterogeneous electron transfer rate constant (k_0) for TiN = 0.0072 $cm{\cdot}s^{-1}$.

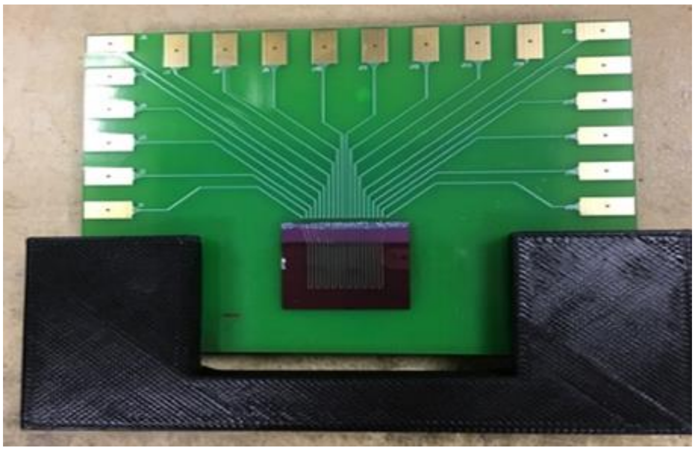

Figure S1. The CMOS sensing chip was fixed on a print circuitry board (PCB). To develop a TiN 3D-NEA-based sensing system, which was then connected to the CHI6116E electrochemical potentiostat (CH Instruments, Inc., Texas, USA) and a personal computer to perform the electrochemical measurements.

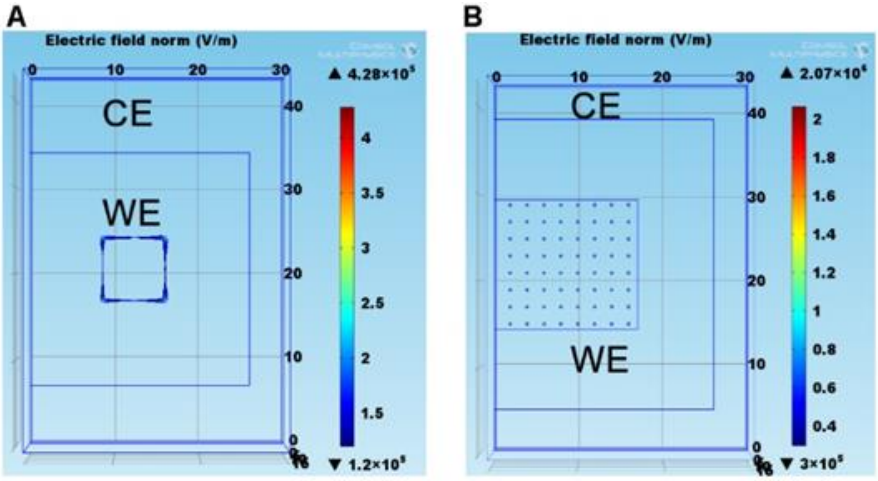

Figure S2. Simulation of electric field distribution of microelectrode and nano-electrode array. The simulation was performed by using COMSOL Multiphysics software. The simulation of electric field distribution of the microelectrode (7.4 × 7.4 μm) (**A**) and an 8 × 8 array of 3D nano-electrode (radius of 0.1 μm) (**B**) in a whole sensing unit view. The gap between nano-electrodes is 2 μm. The highest electric field magnitude (2.07 x 10^6 V/m) in (B) is about 5 folds higher than that (4.28 × 10^5 V/m) in (A).

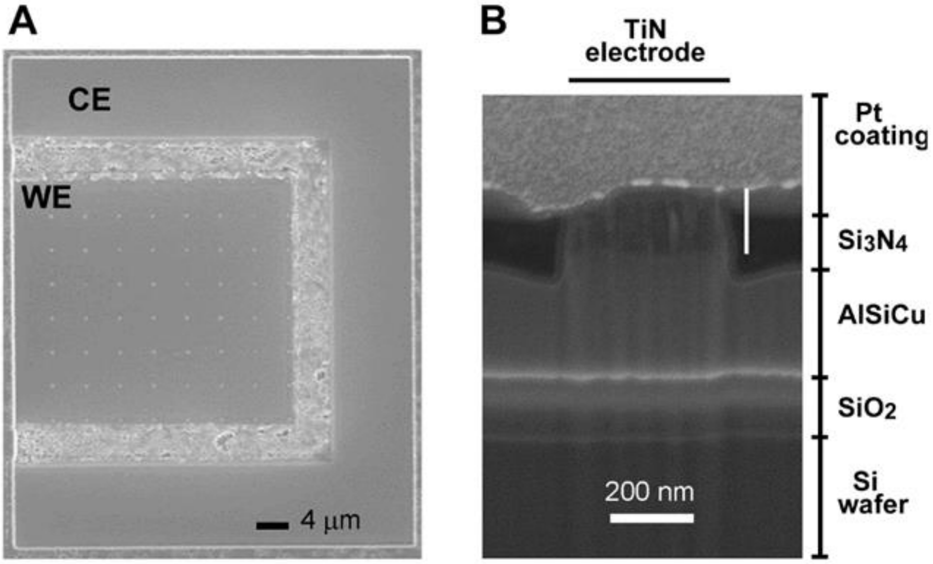

Figure S3. The SEM imaging of the CMOS chip with 3D-NEAL. (**A**) The sensing chip contains a WE of an 8 × 8 array of 3D nano-electrodes of large radius (3D-NEAL) and a counter electrode (CE). The center-to-center distance between nano-electrodes is 4 μm. (**B**) The FIB image of the cross section of the nano-electrode with a radius of 0.175 μm for each electrode. The FIB image of the cross-section of a 3D nano-electrode shows the sequential organization of Si wafer, SiO_2, AlSiCu, TiN electrode and Si_3N_4 structures from the bottom to the top of the device. Pt coating on the top was used to protect the surface of sample from the cutting effect during the FIB imaging. The white vertical bar indicates the TiN electrode.

References

1. Liu, F.; Kolesov, G.; Parkinson, B.A. Time of Flight Electrochemistry: Diffusion Coefficient Measurements Using Interdigitated Array (IDA) *Electrodes. J. Electrochem. Soc*. **2014**, *161*, H3015–H3019.

@2019 by the authors. Licensee MDPI, Basel, Switzerland. This article is an open access article distributed under the terms and conditions of the Creative Commons Attribution (CC BY) license (http://creativecommons.org/licenses/by/4.0/).
